# Constructing arbitral authority in CAS jurisdictional reasoning

**DOI:** 10.3389/fspor.2026.1983695

**Published:** 2026-10-14

**Authors:** Zihan Bai

**Affiliations:** School of Physical Education and Sports, Soochow University, Suzhou, China

**Keywords:** arbitral consent, arbitral jurisdiction, court of arbitration for sport, empirical legal research, sport governance

## Abstract

**Introduction:**

The Court of Arbitration for Sport (CAS) is a central forum for resolving disputes in organized sport, and the legal effect of its jurisdictional provisions depends on how they connect sporting rules, parties, and disputes. The question is how those connections become legally sufficient for jurisdiction.

**Methods:**

Using structured analysis of published decisions and doctrinal interpretation, this article examines how CAS panels make arbitral authority legally operative in jurisdictional reasoning. It draws on a corpus of 172 public CAS decisions, with decision dates between 13 January 2015 and 2 April 2024, and compares the legal connections through which panels establish or deny authority. The analysis distinguishes the procedural route to CAS, the instrument providing for arbitration, and the connection binding the parties to that instrument.

**Results:**

Federation rules, anti-doping provisions, Olympic instruments, specific agreements, and procedural conduct perform different functions within these connections. The comparison shows why participation in organized sport can support jurisdiction in one setting while leaving it unavailable in another.

**Discussion:**

This relational account explains how private sporting rules become arbitral authority and why clarity in jurisdictional drafting matters for athletes, clubs, federations, and anti-doping institutions.

## 1 Introduction

Global sport depends on rules that determine who may compete, which bodies may discipline, how transfers are validated, how anti-doping sanctions are reviewed, and which forum may decide a dispute. These rules are not peripheral to competition. They are the legal infrastructure through which international sport organizes authority across national borders. The Court of Arbitration for Sport (CAS) occupies a central position in that infrastructure. Its awards shape eligibility, anti-doping enforcement, federation governance, Olympic participation, club disputes, transfer consequences, and disciplinary review. For athletes and clubs, the question whether CAS has jurisdiction can decide whether a claim is heard at all. For federations and event organizers, the same question defines the legal reach of their regulatory systems. CAS jurisdiction therefore provides a useful point of entry for studying how global sport governance becomes legally binding (1–6).

Arbitration doctrine usually begins from consent. In the familiar commercial model, parties choose arbitration by contract; jurisdiction is then traced to an arbitration clause or submission agreement. That model remains powerful because it connects arbitral authority to party autonomy and distinguishes arbitration from ordinary adjudication by the state (7, 8). CAS arbitration often works through a more layered legal setting. The individual athlete or club may not have negotiated a separate arbitration clause for the dispute before the panel. Under Article R47 of the CAS Code, panels examine the applicable statutes, regulations, or specific arbitration agreement (27). Licensing conditions, entry forms, and procedural conduct may also establish or confirm the parties' connection to CAS. The doctrinal question is how CAS panels make those materials legally sufficient for jurisdiction.

This question matters because sports arbitration is situated at the intersection of private ordering, transnational regulation, and individual livelihood. A suspension can interrupt a career during the short period in which an athlete is competitive. A transfer or eligibility dispute can alter a club season. An Olympic selection decision may lose practical value once the event has passed. The procedural route to CAS is therefore part of the substantive governance of sport. When a rule directs a party to CAS, it allocates legal authority, sets the available path of review, and structures the time within which rights can be vindicated. The construction of jurisdiction is consequently a matter of sport policy as well as arbitration doctrine.

Existing literature has generated a rich debate on CAS legitimacy, independence, transparency, mandatory arbitration, human rights review, athlete autonomy, and the public visibility of CAS reasoning (9–15, 36). The relationship between sporting participation and arbitral consent is already part of that debate. This article examines how panels apply that relationship to particular parties and disputes, and where the legal connection fails. Its object is the reasoning by which arbitral authority is made operative in the decisions themselves.

The article contributes an empirical legal account of CAS jurisdictional reasoning drawing on 172 public decisions. It identifies the instruments examined by panels and compares the conditions under which participation and regulatory status connect a party to a provision for CAS arbitration. The comparison traces how rules, status, decisions, and procedural conduct combine in the selected decisions.

This comparison places particular weight on the clarity, accessibility, and fairness of the rules that connect participants to CAS. Those rules determine how the transnational dispute-resolution architecture of sport reaches particular parties and disputes.

### 1.1 Literature on consent and jurisdiction in sport

The central disagreement concerns the legal significance of accepting arbitration as a condition of sporting participation. Łukomski (16) questions whether clauses backed by sporting sanctions can amount to a free waiver of access to a court. Rigozzi and Robert-Tissot (17) distinguish the different functions of consent in sports arbitration, including agreement to arbitrate and waiver of judicial review. These accounts show why the validity of an arbitration clause and the freedom of the athlete's choice require separate analysis (34, 35). A panel's recognition of a clause establishes a legal route to arbitration; it does not, by itself, establish that the athlete had a meaningful alternative.

The institutional setting also matters. Baddeley (18) connects the autonomy of sports bodies under Swiss association law with the courts' permissive treatment of sports arbitration. The rules binding an athlete may operate through several organizations and documents. This account places the jurisdictional clause within a broader distribution of private power. The present study examines the corresponding reasoning inside CAS decisions: which document provides for arbitration, how it reaches the party, and whether it covers the dispute. These questions make the legal operation of institutional authority open to comparison.

Olympic disputes expose the difference between being governed by an institution and having an enforceable route to challenge it. Mazzucco and Findlay (19) examine the jurisdictional barriers encountered in challenges to Olympic event selection. Their analysis of Henriques questions the panel's treatment of the Olympic Charter as a basis for an arbitration agreement with athletes. The disagreement concerns how an existing arbitration provision becomes binding between particular parties. It therefore bears directly on a relational account of consent and requires attention to decisions refusing jurisdiction as well as those accepting it.

Football presents a related problem through federation membership, competition entry, and disciplinary review. Łukomski (16) discusses clauses in football statutes and competition regulations that connect participation to CAS arbitration. In the Financial Fair Play context, Sroka (20) examines four major cases and argues that CAS review has affected the effectiveness of UEFA's enforcement regime. The substantive effectiveness of financial regulation and the legal source of appellate authority are distinct questions. For the present analysis, a licensing or financial dispute must be classified by the provision actually authorizing CAS review, rather than by its subject matter. This distinction permits comparison between general statutory appeal clauses and more specific regulatory instruments.

Empirical work on CAS jurisprudence supplies a further point of reference (2). Building on the debates over consent, institutional authority, and access to review, this article examines their operation within panels' jurisdictional reasoning. Article R47, a federation statute, and an Order of Procedure may appear in one decision while performing different legal tasks. Comparing those tasks across regulatory settings explains both access to CAS and its boundaries.

The article proceeds as follows. Section 2 sets out the study design, data source, extraction protocol, and use of descriptive analysis. Section 3 presents the empirical findings and explains the principal legal-basis patterns. Section 4 discusses what those patterns show about governance-based arbitral authority in sport and their implications for policy, athlete protection, and arbitration theory. Section 5 concludes by identifying the central doctrinal lesson of the study.

## 2 Materials and methods

### 2.1 Research design and legal materials

This article uses empirical legal research to study published CAS awards as legal texts (21, 22). The method combines structured documentary analysis, descriptive counts, and doctrinal interpretation. The descriptive component records the composition of the corpus, while the legal component asks what the cited instruments do in the tribunal's reasoning. The analysis explains how CAS panels justify authority in the published awards and why that reasoning matters for sport governance.

The unit of analysis is the published CAS decision. Consolidated docket numbers represented by a single decision are recorded together. The corpus includes jurisdictional and partial decisions as well as final awards; the analysis concerns the panel's reasons for exercising or declining authority in the proceeding before it.

The study draws on decisions in the official CAS jurisprudence database (33). Public decisions provide the reasons available to athletes, counsel, federations, national courts, researchers, and policymakers. Table 1 summarizes the source, decision-date window, and extraction fields.

**Table 1:** Dataset and extraction design.

Item	Description
Data source	Official CAS jurisprudence database.
Retained corpus	172 public CAS decision records.
Search dates	22 July 2026; supplementary search 23 July 2026.
Decision dates	Search window 2015–2025; retained decisions 13 January 2015 to 2 April 2024.
Unit	Published decision; consolidated docket numbers recorded together.
Extraction fields	Database label, adjudicatory role, specific jurisdictional instruments, passage and source location.
Search record	Recoverable collection counts in S1; case-level instrument register in S2.

Official CAS database and collection register (Supplementary Note S1). The table distinguishes the search settings from the dates of the retained decisions.

### 2.2 Case collection and inclusion criteria

The corpus was assembled through the official CAS database's jurisdiction-related keyword categories within a decision-date search window of 1 January 2015 to 31 December 2025. The initial collection was conducted on 22 July 2026 and identified 166 distinct database records. The keyword categories included CAS jurisdiction, arbitration agreement, lack of jurisdiction, and their associated subcategories, together with Kompetenz-Kompetenz. A supplementary search on 23 July 2026 for decisions dated in 2024–2025 returned eight records, of which two were already present and six were added. The retained corpus contains 172 records, with decision dates from 13 January 2015 to 2 April 2024. Supplementary Note S1 reports the recoverable search counts and their basis.

The corpus comprises indexed public decisions containing a passage addressing CAS's own authority, including decisions accepting or refusing jurisdiction and decisions recording uncontested jurisdiction. Jurisdictional orders and partial awards are included where they address that question. A record addressing only a federation's internal competence would not satisfy this criterion. The 172-record register is retained for the instrument-level analysis in Supplementary Table S2.

The retained initial register records 307 keyword–record associations representing 166 distinct decisions, with 141 repeated associations across keywords. All 166 decisions were retained. The supplementary retrieval contributed six further decisions after two duplicate records were removed. Supplementary Note S1 provides the collection flow, the reconciliation of the aggregate keyword count, and the provenance of the reported counts. The corpus is a purposively assembled collection of public decisions, not an exhaustive set of decisions within the search window.

### 2.3 Extraction protocol

The extraction records case number, decision date, database procedure label, source link, jurisdictional passage, and its location. The instrument register identifies the concrete provisions discussed for CAS jurisdiction and separates them from rules concerning the earlier tribunal or the merits. It also records the adjudicatory role exercised or sought in the current proceeding. The detailed case comparison separately identifies the panel's conclusion and the connection between the provision and the parties.

The extraction preserves the jurisdictional passage and its location alongside the descriptive fields. Legal interpretation distinguishes the tribunal's finding from submissions, quotations of earlier decisions, and discussion of another body's competence.

The analysis distinguishes three elements: the procedural route to CAS; the specific statute, regulation, or agreement providing for arbitration; and the connection between that instrument and the parties or dispute. Article R47 is recorded as an appellate provision, not as an independent authorization supplied by a sports body. The relevant instrument is identified by name and provision, including its applicable version where stated in the decision. A contract incorporating regulations and an Order of Procedure confirming jurisdiction are recorded separately, because they may operate alongside a statutory appeal clause.

### 2.4 Pattern identification and use of descriptive tables

Table 2 relates the database labels to the adjudicatory role described in the decisions. Table 3 reports the instrument families examined within each role; a decision may appear in more than one row. The counts include specifically tested provisions whose applicability was rejected or left undecided, and therefore describe the instruments examined in jurisdictional reasoning. Generic quotations of Article R47, Swiss PILA Article 186, or earlier cases do not establish an instrument category. Table 4 identifies the provisions, party connections, and findings in the detailed comparison.

**Table 2:** Database labels and adjudicatory roles in the retained corpus.

Database label	Records	Role identified in decision	Records
Appeal Procedure	136	Appeal/direct sole-instance	132/4
Ordinary Procedure	25	Direct sole-instance	25
Ad hoc Procedure	10	Olympic *ad hoc*/Olympic ADD	7/3
Anti-doping Procedure (first instance)	1	CAS ADD	1
Total	172	All roles	172

Official CAS database labels and decision-level coding (Supplementary Table S2; *n* = 172). The comparison distinguishes the database label from the adjudicatory role.

**Table 3:** Instruments examined within each adjudicatory role.

Instrument or route	Appeal *n* = 132	Direct *n* = 29	Olympic *ad hoc n* = 7	ADD *n* = 4
Statutes and constitutions	88 (66.7)	0 (0.0)	0 (0.0)	0 (0.0)
Dispute-resolution and transfer regulations	9 (6.8)	0 (0.0)	0 (0.0)	0 (0.0)
Disciplinary and ethics instruments	13 (9.8)	0 (0.0)	0 (0.0)	0 (0.0)
Competition, selection and admission instruments	9 (6.8)	0 (0.0)	0 (0.0)	0 (0.0)
Anti-doping rules and codes	26 (19.7)	27 (93.1)	0 (0.0)	4 (100.0)
National anti-doping legislation	3 (2.3)	0 (0.0)	0 (0.0)	0 (0.0)
Olympic Charter	6 (4.5)	0 (0.0)	7 (100.0)	3 (75.0)
Specific contractual or written submissions	15 (11.4)	2 (6.9)	0 (0.0)	0 (0.0)
Participation and eligibility undertakings	3 (2.3)	0 (0.0)	0 (0.0)	0 (0.0)
Procedural acceptance as articulated route	8 (6.1)	0 (0.0)	0 (0.0)	0 (0.0)

Instrument-level coding in Supplementary Table S2 (*n* = 172). Percentages describe the instruments examined within each role.

Values are n (% within role). Categories overlap and columns do not sum to 100%. Counts identify concrete instruments considered for the current proceeding, including rejected or undecided routes; they do not count valid agreements or individually negotiated consent. ADD combines three Olympic ADD decisions and one CAS ADD decision. Routine confirmations of a separately identified source are not counted as an additional procedural-acceptance route.

**Table 4:** Instruments and jurisdictional connections in the detailed case comparison.

Decision and paragraphs	Instrument and connection	Finding
2015/A/3889 (38) 5.1–5.8	RFU Statutes 47 (1) and Dispute Resolution Regulations 53 (2); agent licence; Order of Procedure confirms.	Jurisdiction accepted.
2017/A/4949 (43) 61–65; 79–84	Current appeal: IAAF Rule 42 and R47. Earlier sole-instance authority assessed separately under Rule 38.3.	Appeal jurisdiction accepted; prior procedural route distinguished.
2015/A/3970 (39) 50–59	TADR and IAAF appeal provisions; support personnel for national-level athletes; no applicable direct CAS route.	Jurisdiction denied.
OG 18/004 (42) 5.1–5.16; 6.1	Olympic Charter 61.2 and *ad hoc* Article 1; dispute arose outside the ten-day window.	Ad hoc jurisdiction denied.
2018/O/5754 (41) 27; 99–100	Specific arbitration agreement; direct sole-instance submission using appeal procedural provisions.	Jurisdiction accepted.
2015/A/4254 (40) 46–51	R55 and PILA 186 (2); one late objection; the other respondent expressly withdrew its objection.	Jurisdiction accepted.

The six CAS decisions and paragraph references listed above. The comparison connects each provision and party relationship to the jurisdictional finding.

The within-role comparisons distinguish the documents through which access to CAS is claimed or established. A contractual submission is counted only where a specific clause or written instrument is identified; its presence does not establish that arbitration was individually negotiated. Participation and eligibility forms are recorded separately. Procedural acceptance is identified where it supplies the articulated route to jurisdiction, rather than merely confirming a separately identified rule. Supplementary Table S2 provides the case-level coding decisions and locations.

The analysis distinguishes CAS jurisdiction from the competence of the body whose decision is challenged. In CAS 2019/A/6569 (46), for example, the panel identifies FIFA Statutes Article 58(1) as the source of CAS review and addresses the contractual objection to FIFA's competence on the merits (paras 65–68). The employment clause is consequently not counted as a separate source of CAS authority. This distinction also applies where an appellate panel reviews an earlier CAS sole-instance decision.

### 2.5 Research scope

The corpus is selected through the public database's indexing and publication practices. Its procedure distribution describes the retained public decisions. Comparisons concern how identified instruments operate within each procedural setting; they do not estimate the balance between regulatory and separately agreed jurisdiction in confidential Ordinary Division proceedings (14).

The instrument counts record the provisions examined; the detailed case analysis identifies whether a panel accepted them as a basis for jurisdiction. Where a panel leaves a proposed regulatory route undecided because the parties accepted jurisdiction, the register retains that distinction. Agreements are coded where they are identified in the published reasoning.

## 3 Results

### 3.1 The jurisdictional landscape

The retained corpus contains 172 public CAS decisions. The database labels 136 as Appeal Procedure, 25 as Ordinary Procedure, 10 as *ad hoc* Procedure, and one as Anti-doping Procedure (first instance). The decisions identify 132 appellate proceedings, 29 direct sole-instance proceedings, seven Olympic *ad hoc* proceedings, and four Anti-Doping Division proceedings (Table 2). Four records labelled as appeals expressly apply IAAF Rule 38.19 to direct sole-instance adjudication. Three records labelled *ad hoc* belong to the Olympic Anti-Doping Division. Appeals account for 132 of 172 decisions (76.7%); this composition makes comparisons within each role necessary when interpreting the instrument counts in Table 3.

The appellate decisions examine federation or committee statutes in 88 of 132 records (66.7%), disciplinary and ethics instruments in 13 (9.8%), dispute-resolution or transfer regulations in nine (6.8%), and competition, selection, or admission instruments in nine (6.8%). These categories overlap. Their distribution identifies the specific documents examined within the appellate procedure. The register also separates provisions tested unsuccessfully from those supporting the detailed jurisdictional findings.

The direct sole-instance group is concentrated in anti-doping referrals: 27 of 29 decisions discuss anti-doping rules, while two identify specific written submission instruments. The latter are CAS 2018/O/5754 (41) and CAS 2018/O/5830 (45). In the second case, the panel found an agreement in an unsigned MoU and the parties' subsequent written communications (paras 160–161). Within this anti-doping setting, the comparison shows how a regulatory referral and a specific submission each bring the dispute before CAS.

The central comparison concerns the connection between the source and the dispute. A rule may provide for CAS arbitration while leaving a particular claimant, decision, or period outside its scope. Conversely, a specific agreement may confer authority even where the proceeding uses rules associated with another procedural division. Table 4 identifies the provisions and outcomes underlying this comparison.

### 3.2 Article R47 and sports-body rules as the central appellate channel

In appellate decisions, panels examine Article R47 of the CAS Code and the rules of a sports body. Article R47 functions as a procedural threshold that directs the panel to identify an appealable decision and to determine whether the applicable statutes or regulations provide for an appeal to CAS. The provision therefore links the CAS Code to the regulatory instruments of international and national sports bodies. In this pattern, arbitral authority is produced by the interaction of CAS procedure and sport-specific governance rules (1, 23, 24).

The practical effect is significant. Article R47 allows CAS appellate jurisdiction to operate across many sports without requiring each dispute to be accompanied by a separate negotiated arbitration agreement. A federation statute, disciplinary code, transfer regulation, licensing rule, or specific arbitration agreement may supply the route to CAS. The panel then examines whether that route covers the parties and the decision under challenge (37). The legal inquiry is concrete: the rule must exist, the decision must be appealable, and the appellant must fall within the regulatory framework that grants access to CAS.

CAS 2015/A/3889 (38) illustrates the structure. The panel located jurisdiction through Article R47, Article 47(1) of the Russian Football Union Statutes, and Article 53(2) of its Dispute Resolution Regulations (paras 5.1–5.4). The latter provision made the Players' Status Committee decision appealable “only to CAS”. The appellant's RFU agent licence supplied the relevant institutional connection, while the signed Order of Procedure further confirmed jurisdiction (paras 5.6–5.8). The statutory and regulatory provisions supplied the appeal route; the licence connected the agent to that framework. The Order of Procedure performed a confirming function. The case shows why these instruments should be identified separately even when they support the same jurisdictional conclusion.

The negative cases identify the limits of the same route. A provision must cover the parties and the decision under challenge. Jurisdiction, admissibility, and standing are recorded according to the panel's treatment of each issue, rather than merged whenever an appeal fails. In particular, dismissal of an appeal on its merits does not establish that CAS lacked jurisdiction.

The statutory route can also reach a non-member where the clause is drafted broadly. In CAS 2016/A/4787 (44), the panel applied UEFA Statutes Article 62 to JFA as a party directly affected by a UEFA decision, rejecting the argument that only members could appeal (paras 94–99). Membership therefore supplies one possible connection, but the wording of the applicable clause determines whether it is necessary.

### 3.3 Anti-doping rules and direct regulatory authority

Anti-doping decisions illustrate both appellate and direct sole-instance jurisdiction. The analysis distinguishes rules providing for a first-instance or sole-instance determination from provisions authorizing an appeal. This pattern reflects the institutional density of anti-doping governance. Anti-doping rules identify the athlete, the responsible anti-doping organization, the testing or results-management framework, available sanctions, and review routes. CAS jurisdiction is often constructed within that regulatory architecture through provisions applicable to the athlete and the relevant sports body (12).

The legal basis in these awards frequently turns on status. An athlete becomes subject to anti-doping rules through federation membership, event participation, licensing, registration, or inclusion within a sport regulatory system. Once that status is established, the rules allocate competence among disciplinary bodies and CAS (31, 32). The panel's task is to trace that allocation. It asks whether the relevant anti-doping rules apply to the athlete, whether the body issuing the decision had authority, whether the appeal route is available, and whether CAS is designated as the forum for review or first-instance determination.

CAS 2017/A/4949 (43) makes this distinction explicit. The appeal panel derived its own jurisdiction from Article R47 and IAAF Rule 42 (paras 61–65), then separately reviewed the first-instance arbitrator's authority. It held that Rule 38.3's reference to appeal procedure concerned the handling of that earlier case, rather than the threshold requirements for appellate jurisdiction (paras 79–84). Requiring an earlier decision would frustrate a rule designed to operate when a national body had not supplied one. The decision therefore distinguishes the procedural rules used by a tribunal from the regulatory provision authorizing it to act.

These decisions show how panels connect regulatory status to a specific jurisdictional provision. Anti-doping law operates as a regulatory regime to which athletes are subject as a condition of participation in organized sport. In these decisions, panels explain that structure through jurisdictional reasoning: they identify the applicable code, connect it to the athlete or organization, and determine whether the dispute falls within the assigned CAS competence.

CAS 2015/A/3970 (39) shows where the connection fails. The appellant was support personnel for national-level athletes, and the applicable rules provided a domestic appeal route (paras 50–52). He argued that defects in the independence of that forum should allow direct recourse to CAS. The panel rejected that inference: CAS could not be treated as a “default appeal tribunal” (para. 55). Neither the applicable Turkish anti-doping rules nor a specific agreement conferred jurisdiction over his appeal. The panel accordingly declined jurisdiction without deciding timeliness (paras 58–59). Regulatory status was decisive because it determined the available appeal route; a concern about the domestic forum did not itself create another one.

The anti-doping pattern also has policy significance. It shows that the enforceability of global anti-doping governance depends on transparent connections between athletes, federations, anti-doping organizations, and CAS. If those connections are clear, CAS can provide a transnational forum for consistent review. If they are obscure, the jurisdictional stage becomes a site of avoidable delay and contestation. For athletes, this clarity affects access to review during the period when a sanction has immediate career consequences.

### 3.4 Olympic and *ad hoc* jurisdiction

Olympic *ad hoc* jurisdiction is temporary, event-centered, and closely tied to the governance needs of the Games. It is commonly reasoned through the Olympic Charter, the CAS Arbitration Rules for the Olympic Games, entry materials, or related Olympic instruments. The result is a jurisdictional model designed for urgency: disputes must be resolved quickly enough to preserve the practical value of competition-related rights.

Rule 61 of the Olympic Charter and Article 1 of the CAS *ad hoc* rules are central to this pattern (28, 29). They create a route to CAS for disputes connected to the Olympic Games and subject to temporal limits. The temporal requirement is not a technical detail. It defines the boundary between ordinary sports disputes and those requiring immediate Olympic adjudication. The panel must determine whether the dispute arises during the Games or within the specified period before the Opening Ceremony, and whether the subject matter is sufficiently connected to Olympic participation.

CAS *ad hoc* Division OG 18/004 (42) illustrates the temporal boundary. Applying Rule 61.2 of the Olympic Charter and Article 1 of the *ad hoc* rules, the panel treated the ten-day condition as a question of jurisdiction (paras 5.1–5.5). The applicants relied on an IOC communication of 2 February 2018. The panel found that the dispute had already arisen on 19 January, when the non-selection list was communicated, and therefore fell outside the required period (paras 5.11–5.16). It declined jurisdiction. The Olympic connection identified the relevant framework, but it did not remove that framework's temporal condition. The result turns on when the dispute arose, rather than simply when the application was filed.

This pattern contributes to the article's broader claim because it shows consent operating through event participation and Olympic status. Athletes enter a legal order created for a specific competition. Within that order, jurisdiction is constructed through the Olympic instruments that govern participation and dispute resolution. The panel's reasoning transforms event participation into access to a specialized arbitral forum. At the same time, the temporal limits prevent that transformation from expanding beyond the event-centered function of the *ad hoc* division.

### 3.5 Agreement, procedural conduct, and competence-competence

Separately identifiable agreements provide a useful comparison within anti-doping proceedings. In CAS 2018/O/5754 (41), the parties signed an agreement submitting the dispute directly to CAS without a prior RUSADA hearing. The agreement provided that the case would proceed before the Ordinary Division as a sole-instance case while applying provisions used for appeal arbitration (para. 27). The panel held that jurisdiction was “expressly contemplated by the Arbitration Agreement” (paras 99–100). Neither the anti-doping subject matter nor the use of appeal procedure made this a regulatory appeal. The source of authority was the agreement submitting this dispute to CAS.

Procedural conduct provides another connection. In CAS 2015/A/4254 (40), El Dakhlia addressed the merits in its answer and raised its jurisdictional objection later. The panel applied Article R55 and Swiss PILA Article 186(2), finding that the objection should have been made “together with the Answer at the latest” (paras 46–49). EFA had initially objected but expressly withdrew its objection at the hearing (para. 50). The panel consequently accepted jurisdiction (para. 51). The reasoning distinguishes late objection from express confirmation; neither should be counted simply because an Order of Procedure is mentioned elsewhere in a decision.

Swiss arbitration law supplies an important background to this reasoning. CAS has its seat in Switzerland, and Swiss private international law recognizes core doctrines such as competence-competence and rules concerning arbitration agreements and arbitral jurisdiction (25, 30). Panels may refer to Swiss law where a jurisdictional objection requires attention to the tribunal's power to rule on its own competence or to the formal basis of arbitration. The power to determine competence must be distinguished from the rule or agreement conferring jurisdiction over the particular dispute.

These mechanisms show the plural character of CAS jurisdiction. Agreement, procedural conduct, and Swiss-law principles interact with regulatory materials. A party may be brought within the CAS system through federation rules and then confirm the process by signing an Order of Procedure or failing to raise an objection in time. A contract may incorporate sporting rules that point to CAS. A panel may use Swiss competence-competence doctrine to address a challenge to a rule-based jurisdictional route. CAS consent is therefore assembled through multiple legal techniques.

### 3.6 Denied and limited jurisdiction as boundary-setting reasoning

Decisions declining jurisdiction identify which part of the connection is absent. CAS 2015/A/3970 (39) concerns the absence of a CAS appeal route for the appellant's regulatory position; OG 18/004 (42) concerns the temporal reach of the *ad hoc* forum. Both disputes were connected to organized sport, but that connection alone did not satisfy the applicable jurisdictional conditions.

The boundary cases identify the conditions of arbitral authority. CAS jurisdiction depends on a legally operative connection between party, decision, dispute, and rule. Where that connection exists, a panel may construct authority without a separate negotiated arbitration agreement. Where the connection is missing, participation in sport alone is insufficient. The legal chain must be complete. This is why the article describes CAS consent as governance-based rather than automatic. The governance relation supplies a route to jurisdiction only through identifiable legal instruments.

The boundary cases also reveal a practical drafting lesson for sports organizations. Jurisdiction clauses must identify the forum, the decisions subject to appeal, the persons bound, any internal remedies that must be exhausted, and the time limits for bringing the case. Ambiguity at any of these points creates jurisdictional friction. Clear drafting makes the allocation of authority visible. In a system where parties often encounter CAS through rules rather than through negotiated clauses, visibility is a central governance value.

## 4 Discussion

### 4.1 From agreement to governance relation

The examined decisions support a relational account of arbitral authority, consistent with wider accounts of arbitration as a form of transnational governance (26). Traditional arbitration doctrine ties jurisdiction to a discrete act of agreement. The selected decisions locate the relevant act within a broader governance relation. The athlete registers, competes, accepts federation rules, enters an event, becomes subject to anti-doping regulation, or participates in an appeal system. The club affiliates with a federation or participates in competitions governed by sport-specific rules. These statuses become relevant to jurisdiction where the applicable instruments provide a route to CAS. The panel then identifies the instruments that make those consequences operative.

This comparison identifies how consent is evidenced through the instruments governing organized sport. CAS panels continue to search for a legal basis that can justify arbitral authority. They cite rules, agreements, decisions, orders of procedure, statutes, and governing instruments. The comparison identifies how institutional rules and specific agreements supply the relevant jurisdictional connection. Consent is expressed through entry into a governed sporting relationship and through acceptance of the rules attached to that relationship.

Paulsson's account of arbitration emphasizes the centrality of consent to the idea of arbitration (8). CAS practice remains in conversation with that idea, but it demonstrates that consent can be mediated through institutional status. In a governance setting, the decisive legal relationship may be created before the dispute and outside a separately negotiated arbitration clause. The athlete or club becomes positioned within a rule system that includes CAS jurisdiction as part of its architecture. The award then explains how that architecture reaches the dispute before the panel.

This relational construction is especially visible in the Article R47 and anti-doping patterns. Article R47 requires a panel to look outward, from the CAS Code to the rules of the sports body. Anti-doping jurisdiction requires the panel to identify the athlete's regulatory status and the competence allocated by the relevant code. In both settings, the authority of CAS arises from the interaction between CAS procedure and the normative order of sport. The tribunal traces the provisions through which that governance relationship supplies jurisdiction.

### 4.2 Contribution to sport policy and law

The study contributes to sport policy by identifying jurisdiction clauses and appeal routes as governance instruments. They should not be treated as technical boilerplate at the end of federation statutes. They determine where disputes go, how quickly they can be heard, and whether a party has access to a transnational forum. When CAS jurisdiction is built through rules, the quality of those rules affects the quality of dispute resolution. Clarity, accessibility, translation, notice, and consistency become central policy concerns.

This contribution is practical for federations and event organizers. Rules that confer CAS jurisdiction should state who is bound, which decisions are appealable, whether internal remedies must be exhausted, which version of the CAS Code applies, and how time limits run. Anti-doping rules should identify the relationship between national anti-doping organizations, international federations, first-instance bodies, appellate routes, and CAS. Olympic and event-specific instruments should make temporal and substantive boundaries clear. These drafting practices reduce procedural disputes and make authority easier to verify.

The same point matters for athletes and clubs. A party cannot plan litigation strategy or protect its rights if the route to CAS is dispersed across multiple documents that are difficult to locate or understand. Jurisdictional clarity is therefore part of access to justice in sport. It enables parties to know whether CAS is available, when an appeal must be filed, which decision can be challenged, and what procedural steps are required before arbitration. In sports disputes where delay can defeat the practical value of relief, that knowledge helps parties preserve access to review.

The findings also speak to debates about private power in global sport. CAS jurisdiction often gives legal effect to rules produced by sports bodies rather than by states. That structure can support coherence across borders, especially in anti-doping and Olympic disputes. It also places responsibility on sports institutions to draft and apply jurisdictional rules in a manner that can withstand legal scrutiny. The public award is one site where that scrutiny occurs. By reading those awards systematically, this article shows how governance authority is translated into legal authority.

### 4.3 Implications for athletes, clubs, federations, and anti-doping bodies

For athletes, the key implication is that participation in organized sport often carries dispute-resolution consequences long before a dispute arises. Licensing, registration, event entry, and acceptance of anti-doping rules can place an athlete within a jurisdictional framework that later points to CAS. The examined decisions connect regulatory participation to particular arbitration provisions. The strength of that legal connection depends on the applicable rules and the party's position within them. Athlete-facing rules should therefore be drafted and presented as consequential legal instruments.

For clubs, the findings are similarly important. Club disputes often reach CAS through federation statutes, league rules, transfer regulations, disciplinary codes, or contractual instruments that incorporate sport rules. The legal basis may be distributed across several documents. Clubs therefore need to understand CAS jurisdiction as part of regulatory compliance, not only as a litigation issue that arises after a dispute. The awards show that procedural rights and obligations are formed within the same governance structures that organize competition and membership.

For federations, the findings identify a legal design task. A federation that wishes to confer CAS jurisdiction must create a rule chain that is clear, internally consistent, and connected to appealable decisions. The decisions declining jurisdiction distinguish missing authorization from the deliberate limits of a rule. If a rule does not identify CAS, if internal appeal requirements are unclear, or if the decision-maker is not covered by the relevant provision, the jurisdictional pathway may fail. Good jurisdictional drafting protects institutional authority by making the source of that authority legally legible.

For anti-doping bodies, the study shows why harmonization and clarity remain central. Anti-doping enforcement depends on layered relationships among athletes, international federations, national anti-doping organizations, laboratories, disciplinary bodies, WADA, and CAS. The jurisdictional reasoning in the awards makes those relationships visible. CAS authority is strongest where the rules identify the athlete's status, the body's competence, the decision under review, and the available appeal route. The practical value of the anti-doping system depends on that structure being understandable before a dispute reaches CAS.

### 4.4 Implications for arbitration theory

The findings have a broader implication for arbitration theory. Sports arbitration shows that consent can operate through institutional relations as well as bilateral bargains (7, 8, 24). The commercial arbitration model remains essential where parties negotiate a contract and agree to arbitrate disputes arising from that contract. CAS practice demonstrates another setting in which arbitral authority is produced through organized participation in a transnational regulatory order. This is a distinctive legal construction of consent.

The distinction matters because it clarifies the debate. Critiques of mandatory sports arbitration often argue that athlete consent is constrained. Defenses of CAS often point to the rule-based structure of organized sport and the need for uniform dispute resolution. The decisions studied here permit comparison of the legal connections at issue in those debates. The comparison concerns the legal instruments that attach to participation and whether those instruments confer jurisdiction. The analysis therefore exposes the working legal grammar of CAS authority.

This grammar has three recurring features. First, jurisdiction is textual: panels look for rules, statutes, codes, clauses, or procedural provisions. Secondly, jurisdiction is relational: those texts must connect the party, the decision, and the dispute to CAS. Thirdly, jurisdiction is bounded: the rule chain may fail if the relevant decision is absent, internal remedies are not exhausted, temporal limits are not met, or the party falls outside the applicable framework. These features explain how CAS can be both deeply embedded in sport governance and legally dependent on specific jurisdictional instruments.

The theoretical contribution is therefore precise. The comparison explains how a party's position within organized sport becomes relevant to a provision conferring CAS jurisdiction. It identifies the instruments that make that connection operative and the conditions that prevent it from reaching a particular dispute.

### 4.5 Research boundaries and future research

The findings concern the jurisdictional reasoning published in the selected decisions. The analysis distinguishes the existence of a legal connection from the freedom with which it was accepted. The normative assessment of mandatory arbitration consequently remains distinct from the identification of an operative jurisdictional clause.

Further research can compare jurisdictional reasoning across sports and party positions, including objections raised by athletes, clubs, federations, and anti-doping bodies. Examining national courts' treatment of those reasons would also connect the analysis of CAS authority to judicial review and enforcement.

## 5 Conclusion

This article has examined how CAS panels construct arbitral authority in jurisdictional reasoning. The comparison identifies how connected legal instruments establish authority and where their reach ends. Article R47, federation statutes, anti-doping rules, Olympic instruments, internal appeal requirements, party conduct, and Swiss-law principles form the recurring materials through which panels establish, limit, or deny authority.

The central claim is that participation in organized sport becomes jurisdictionally relevant through identifiable legal instruments. It is made legally operative through rules that identify parties, decisions, procedures, appeal routes, and temporal limits. CAS panels continue to require a jurisdictional basis, and its scope depends on the provisions connecting the party and dispute to CAS. The same comparison can inform further work on sport-specific disputes and judicial review.

This finding matters for sport policy because jurisdiction rules are instruments of governance. They determine access to review and allocate authority across a transnational sporting system. It matters for arbitration theory because it shows how consent can be maintained as a doctrinal requirement while being evidenced through institutional status and regulatory participation. CAS awards therefore reveal a distinctive legal technology of global sport: private rules are translated into arbitral authority through jurisdictional reasoning.

## Data Availability

The original contributions presented in the study are included in the article/Supplementary Material, further inquiries can be directed to the corresponding author.
