# Supplementary material for: Constructing arbitral authority in CAS jurisdictional reasoning
Source: Front Sports Act Living. 2026 Oct 14;8:1983695. doi: 10.3389/fspor.2026.1983695 (PMC13715135; doi:10.3389/fspor.2026.1983695)
Supplement: Supplementary file 1 [file Datasheet1.pdf]

Supplementary Note S1

Corpus and collection dates

The study examines 172 public CAS decisions, including jurisdictional orders and partial decisions. Each record has a distinct database identifier and a source link. Supplementary Table S2 provides the corresponding instrument codes and locations in the decisions.

The initial search was conducted on 22 July 2026 using the official database keyword categories listed below, with decision dates set from 1 January 2015 to 31 December 2025. A supplementary search on 23 July 2026 covered decision dates in 2024–2025. The retained decisions are dated from 13 January 2015 to 2 April 2024. These are the earliest and latest dates in the retained corpus; Decision dates are distinct from search dates and publication dates.

Collection counts

The saved initial register contains 307 keyword–decision associations involving 166 distinct decisions. A decision can appear under more than one keyword, accounting for 141 repeated associations. These figures describe the saved register, not the total number of raw search results. All 166 initial decisions were retained. The supplementary search returned eight records, of which two were already present, giving six additions and a corpus of 172 decisions.

Collection measure	Number	Meaning
Recorded keyword–decision associations	307	Entries across the keyword categories below
Repeated associations	141	Additional keyword entries for the same decisions
Distinct initial decisions	166	All retained in the corpus
Supplementary records returned	8	Two already present in the initial register
Supplementary additions	6	Distinct additional decisions
Final corpus	172	Distinct database records

Decisions were retained where the public text contained an identifiable discussion of CAS jurisdiction, including acceptance, rejection and uncontested jurisdiction. Preliminary screening flags did not constitute exclusion decisions. Complete raw-result counts and reason-coded exclusions before the saved candidate register are unavailable. A contemporaneous total of published decisions for the search window is also unavailable, so a sample-to-database percentage cannot be established.

Keyword categories

Counts are reconstructed from the retained keyword memberships. A decision may contribute to several categories.

Official keyword	Recorded associations
CAS jurisdiction	132
CAS jurisdiction (by reference)	7
CAS jurisdiction (submission)	1
Kompetenz-Kompetenz	16
arbitration agreement	30

Official keyword	Recorded associations
arbitration agreement (autonomy)	2
arbitration agreement (binding non-signatories)	3
arbitration agreement (by reference)	7
arbitration agreement (definition)	0
arbitration agreement (essential points)	5
arbitration agreement (extension to third parties)	4
arbitration agreement (form)	7
arbitration agreement (interpretation)	10
arbitration agreement (lack)	6
arbitration agreement (scope)	3
arbitration agreement (tacit)	0
arbitration agreement (validity)	10
arbitration agreement (waiver)	1
autonomy of the arbitration agreement	0
interpretation of an arbitration agreement/clause	9
lack of jurisdiction	23
lack of jurisdiction (of FIFA instances)	15
lack of jurisdiction (plea of)	15
termination of the arbitration agreement	1
validity of an arbitration agreement	0
Total	307

Procedure labels and adjudicatory roles

Official database label	Records	Percent of retained corpus
Appeal Procedure	136	79.1
Ad hoc Procedure	10	5.8
Ordinary Procedure	25	14.5
Anti-doping Procedure (first instance)	1	0.6

Four records labelled Appeal Procedure—2016/A/4480, 2016/A/4486, 2016/A/4487, and 2016/A/4655—expressly describe direct sole-instance adjudication under IAAF Rule 38.19. Three records labelled Ad hoc Procedure—OG AD 16/001, OG AD 18/003-PA, and OG AD 18/005-PA—concern the Olympic Anti-Doping Division. The role counts are 132 appeals, 29 direct sole-instance proceedings, seven Olympic ad hoc proceedings, and four ADD proceedings (three Olympic ADD and one CAS ADD). Roles include the jurisdiction sought in proceedings where the tribunal declined authority.

Coverage and coding

The corpus supports comparison of jurisdictional reasoning in the selected decisions. It is not an exhaustive collection for the search window. A September 2026 retrieval identified additional records, including one within the retained date range; these are outside the reported 172-decision corpus. The retained records do not establish

the reason for this retrieval difference. Related proceedings may also share legal reasoning and are not treated as statistically independent observations.

Retained case register

Dates below are the database decision dates. "Initial" identifies the 22 July collection and "Supplement" the six additions from 23 July. Coding and passage locations are provided in Supplementary Table S2.

No.	Official decision	Database decision date	Procedure label	Collection
1	2013/A/3364	13 January 2015	Appeal Procedure	Initial
2	2014/A/3639	8 April 2015	Appeal Procedure	Initial
3	2014/A/3695	13 April 2015	Appeal Procedure	Initial
4	2014/A/3786	4 May 2015	Appeal Procedure	Initial
5	2014/A/3787	4 May 2015	Appeal Procedure	Initial
6	2015/A/3889	9 June 2015	Appeal Procedure	Initial
7	2014/A/3565	1 July 2015	Appeal Procedure	Initial
8	2014/A/3759-PA	24 July 2015	Appeal Procedure	Initial
9	2015/A/3879	14 August 2015	Appeal Procedure	Initial
10	2015/A/4174	11 September 2015	Appeal Procedure	Initial
11	2014/A/3561 & 3614	15 November 2015	Appeal Procedure	Initial
12	2015/A/3970	17 November 2015	Appeal Procedure	Initial
13	2015/A/3971	17 November 2015	Appeal Procedure	Initial
14	2015/A/4024	17 November 2015	Appeal Procedure	Initial
15	2015/A/3910	20 November 2015	Appeal Procedure	Initial
16	2014/A/3868	23 November 2015	Appeal Procedure	Initial
17	2014/A/3869	23 November 2015	Appeal Procedure	Initial
18	2015/A/3959	27 November 2015	Appeal Procedure	Initial
19	2015/A/3896	10 December 2015	Appeal Procedure	Initial
20	2015/A/4213	5 January 2016	Appeal Procedure	Initial
21	2014/A/3852	11 January 2016	Appeal Procedure	Initial
22	2012/A/2877	18 January 2016	Appeal Procedure	Initial
23	2015/A/3946	28 January 2016	Appeal Procedure	Initial
24	2015/A/4254	1 February 2016	Appeal Procedure	Initial
25	2015/A/4195	15 February 2016	Appeal Procedure	Initial
26	2015/A/4255	29 March 2016	Appeal Procedure	Initial
27	2015/A/4083	6 April 2016	Appeal Procedure	Initial
28	2014/A/3776	27 April 2016	Appeal Procedure	Initial
29	2015/A/4288	28 April 2016	Appeal Procedure	Initial
30	2015/A/4335	13 May 2016	Appeal Procedure	Initial
31	2016/A/4441	27 June 2016	Appeal Procedure	Initial
32	2015/A/4215	29 June 2016	Appeal Procedure	Initial
33	2016/A/4615	5 July 2016	Appeal Procedure	Initial
34	2015/A/4280	11 July 2016	Appeal Procedure	Initial

No.	Official decision	Database decision date	Procedure label	Collection
35	2015/A/4328	13 July 2016	Appeal Procedure	Initial
36	2015/A/4351	13 July 2016	Appeal Procedure	Initial
37	2016/A/4703	21 July 2016	Appeal Procedure	Initial
38	2016/A/4657	22 July 2016	Appeal Procedure	Initial
39	OG AD 16/001	14 August 2016	Ad hoc Procedure	Initial
40	2015/A/4172	17 August 2016	Appeal Procedure	Initial
41	2015/A/4309	21 September 2016	Appeal Procedure	Initial
42	2015/A/4266	7 October 2016	Appeal Procedure	Initial
43	2016/O/4454	13 October 2016	Ordinary Procedure	Initial
44	2016/O/4455	13 October 2016	Ordinary Procedure	Initial
45	2016/O/4456	13 October 2016	Ordinary Procedure	Initial
46	2016/O/4457	13 October 2016	Ordinary Procedure	Initial
47	2016/O/4465	13 October 2016	Ordinary Procedure	Initial
48	2016/A/4654	25 November 2016	Appeal Procedure	Initial
49	2016/O/4504	23 December 2016	Ordinary Procedure	Initial
50	2016/O/4702	6 January 2017	Ordinary Procedure	Initial
51	2016/A/4450	24 January 2017	Appeal Procedure	Initial
52	2016/A/4697	3 February 2017	Appeal Procedure	Initial
53	2016/A/4477	9 February 2017	Appeal Procedure	Initial
54	2016/O/4575	10 March 2017	Ordinary Procedure	Initial
55	2016/A/4480	7 April 2017	Appeal Procedure	Initial
56	2016/A/4486	7 April 2017	Appeal Procedure	Initial
57	2016/A/4487	7 April 2017	Appeal Procedure	Initial
58	2016/A/4655	7 April 2017	Appeal Procedure	Initial
59	2016/A/4777	21 April 2017	Appeal Procedure	Initial
60	2016/O/4853	21 April 2017	Ordinary Procedure	Initial
61	2016/O/4854	21 April 2017	Ordinary Procedure	Initial
62	2016/A/4473	1 May 2017	Appeal Procedure	Initial
63	2016/A/4700	15 May 2017	Appeal Procedure	Initial
64	2016/O/4883	17 May 2017	Ordinary Procedure	Initial
65	2016/A/4651	19 May 2017	Appeal Procedure	Initial
66	2016/A/4817-IA	1 June 2017	Appeal Procedure	Initial
67	2016/O/4682	22 June 2017	Ordinary Procedure	Initial
68	2016/O/4683	22 June 2017	Ordinary Procedure	Initial
69	2014/A/3838	13 July 2017	Appeal Procedure	Initial
70	2017/A/4949	18 July 2017	Appeal Procedure	Initial
71	2017/O/5039	18 August 2017	Ordinary Procedure	Initial
72	2017/O/4978	25 August 2017	Ordinary Procedure	Initial
73	2016/A/4787	28 September 2017	Appeal Procedure	Initial
74	2016/A/4839	6 October 2017	Appeal Procedure	Initial

No.	Official decision	Database decision date	Procedure label	Collection
75	2017/A/4947	6 October 2017	Appeal Procedure	Initial
76	2017/A/5057	9 October 2017	Appeal Procedure	Initial
77	2016/A/4770	20 October 2017	Appeal Procedure	Initial
78	2017/A/5065	25 October 2017	Appeal Procedure	Initial
79	2017/A/5133	24 November 2017	Appeal Procedure	Initial
80	2017/A/5072	28 November 2017	Appeal Procedure	Initial
81	2017/A/5128	14 December 2017	Appeal Procedure	Initial
82	2017/A/5333	17 January 2018	Appeal Procedure	Initial
83	OG 18/004	8 February 2018	Ad hoc Procedure	Initial
84	OG 18/005	8 February 2018	Ad hoc Procedure	Initial
85	OG AD 18/003-PA	22 February 2018	Ad hoc Procedure	Initial
86	OG AD 18/005-PA	24 February 2018	Ad hoc Procedure	Initial
87	2017/A/5187	2 March 2018	Appeal Procedure	Initial
88	2017/O/5330	8 March 2018	Ordinary Procedure	Initial
89	2017/A/5054	16 April 2018	Appeal Procedure	Initial
90	2017/O/5376	27 April 2018	Ordinary Procedure	Initial
91	2017/O/5389	27 April 2018	Ordinary Procedure	Initial
92	2017/O/5331	3 May 2018	Ordinary Procedure	Initial
93	2017/O/5332	3 May 2018	Ordinary Procedure	Initial
94	2017/A/5459	11 May 2018	Appeal Procedure	Initial
95	2017/A/5421	14 May 2018	Appeal Procedure	Initial
96	2017/A/5131	7 June 2018	Appeal Procedure	Initial
97	2017/A/5209	28 June 2018	Appeal Procedure	Initial
98	2017/A/5200	10 July 2018	Appeal Procedure	Initial
99	2018/A/5658	7 September 2018	Appeal Procedure	Initial
100	2017/A/5324	31 October 2018	Appeal Procedure	Initial
101	2017/A/5356	31 October 2018	Appeal Procedure	Initial
102	2018/A/5586	16 November 2018	Appeal Procedure	Initial
103	2018/A/5664	4 December 2018	Appeal Procedure	Initial
104	2018/A/5624	17 December 2018	Appeal Procedure	Initial
105	2018/A/5628	21 December 2018	Appeal Procedure	Initial
106	2018/A/5661	24 December 2018	Appeal Procedure	Initial
107	2018/A/5647	28 December 2018	Appeal Procedure	Initial
108	2017/A/5475	21 January 2019	Appeal Procedure	Initial
109	2018/A/5520	24 January 2019	Appeal Procedure	Initial
110	2018/A/5854	31 January 2019	Appeal Procedure	Initial
111	2017/O/5398	1 February 2019	Ordinary Procedure	Initial
112	2018/A/5782	28 February 2019	Appeal Procedure	Initial
113	2018/A/5659	29 March 2019	Appeal Procedure	Initial
114	2018/O/5822	11 April 2019	Ordinary Procedure	Initial

No.	Official decision	Database decision date	Procedure label	Collection
115	2018/O/5754	26 June 2019	Ordinary Procedure	Initial
116	2018/A/5945	28 June 2019	Appeal Procedure	Initial
117	2018/A/5853	2 July 2019	Appeal Procedure	Initial
118	2017/A/5498	3 July 2019	Appeal Procedure	Initial
119	2019/A/6330	9 July 2019	Appeal Procedure	Initial
120	2018/A/5746	30 July 2019	Appeal Procedure	Initial
121	2018/A/5595	5 August 2019	Appeal Procedure	Initial
122	2018/A/6064	13 August 2019	Appeal Procedure	Initial
123	2019/A/6131	14 August 2019	Appeal Procedure	Initial
124	2018/A/6052	19 August 2019	Appeal Procedure	Initial
125	2018/A/5545	23 August 2019	Appeal Procedure	Initial
126	2018/A/5868	5 September 2019	Appeal Procedure	Initial
127	2018/A/6025	16 December 2019	Appeal Procedure	Initial
128	2019/A/6112	27 January 2020	Appeal Procedure	Initial
129	2019/A/6225	3 February 2020	Appeal Procedure	Initial
130	2019/A/6274	3 February 2020	Appeal Procedure	Initial
131	2018/A/5982	20 February 2020	Appeal Procedure	Initial
132	2019/A/6636	8 April 2020	Appeal Procedure	Initial
133	2020/A/6781	21 July 2020	Appeal Procedure	Initial
134	2019/A/6226	4 August 2020	Appeal Procedure	Initial
135	2019/A/6312	4 August 2020	Appeal Procedure	Initial
136	2018/O/5830	5 August 2020	Ordinary Procedure	Initial
137	2020/A/6820	6 October 2020	Appeal Procedure	Initial
138	2019/A/6569	2 November 2020	Appeal Procedure	Initial
139	2020/A/6933	26 January 2021	Appeal Procedure	Initial
140	2020/A/6986	6 April 2021	Appeal Procedure	Initial
141	2020/A/6987	6 April 2021	Appeal Procedure	Initial
142	2020/A/6988	6 April 2021	Appeal Procedure	Initial
143	2021/A/8031	5 July 2021	Appeal Procedure	Initial
144	OG 20/002	18 July 2021	Ad hoc Procedure	Initial
145	OG 20/006 & 008	27 July 2021	Ad hoc Procedure	Initial
146	OG 20/010 & 011	2 August 2021	Ad hoc Procedure	Initial
147	OG 22/002	28 January 2022	Ad hoc Procedure	Initial
148	OG 22/008 & 009 & 010	14 February 2022	Ad hoc Procedure	Initial
149	2021/A/8077	16 February 2022	Appeal Procedure	Initial
150	2021/ADD/14	21 March 2022	Anti-doping Procedure (first instance)	Initial
151	2021/A/8403	20 April 2022	Appeal Procedure	Initial
152	2021/A/8413	10 June 2022	Appeal Procedure	Initial
153	2021/A/7636	27 June 2022	Appeal Procedure	Initial

No.	Official decision	Database decision date	Procedure label	Collection
154	2019/A/6594	26 August 2022	Appeal Procedure	Initial
155	2021/A/8417	31 August 2022	Appeal Procedure	Initial
156	2021/A/7915	7 November 2022	Appeal Procedure	Initial
157	2021/A/8008	7 November 2022	Appeal Procedure	Initial
158	2021/A/7775	25 November 2022	Appeal Procedure	Initial
159	2022/A/8621	30 December 2022	Appeal Procedure	Initial
160	2021/A/7996	16 February 2023	Appeal Procedure	Initial
161	2022/A/8700	24 April 2023	Appeal Procedure	Initial
162	2022/A/9351	8 May 2023	Appeal Procedure	Initial
163	2023/A/9518	5 July 2023	Appeal Procedure	Initial
164	2023/O/9401	27 September 2023	Ordinary Procedure	Initial
165	2022/A/8914	9 February 2024	Appeal Procedure	Supplement
166	2023/A/9477	14 February 2024	Appeal Procedure	Initial
167	2023/A/9611	14 February 2024	Appeal Procedure	Supplement
168	2023/A/10093	23 February 2024	Appeal Procedure	Supplement
169	2023/A/10168	29 February 2024	Appeal Procedure	Supplement
170	2022/A/8664	20 March 2024	Appeal Procedure	Initial
171	2022/A/9053	26 March 2024	Appeal Procedure	Supplement
172	2023/A/9757	2 April 2024	Appeal Procedure	Supplement
