# Supplementary material for: Constructing arbitral authority in CAS jurisdictional reasoning
Source: Front Sports Act Living. 2026 Oct 14;8:1983695. doi: 10.3389/fspor.2026.1983695 (PMC13715135; doi:10.3389/fspor.2026.1983695)
Supplement: Supplementary file 2 [file Datasheet2.pdf]

Supplementary Table S2

Instrument coding and adjudicatory role

The register covers 172 decisions and identifies the provisions considered in determining CAS jurisdiction. Coding is non-exclusive: a decision may discuss a statute, a specialized rule, and a contractual submission. A source category identifies a jurisdictional instrument considered, not necessarily an instrument ultimately accepted. Rejected clauses remain visible because they identify the boundaries examined by the tribunal. Generic quotations of R47, competence-competence, and citations to other cases do not by themselves support an instrument code. Rules concerning only FIFA or another earlier tribunal are excluded from the current CAS source classification.

The role field records the adjudication exercised or sought, rather than inferring it from an A/O docket prefix or the database label. The 29 direct sole-instance records include 25 labelled Ordinary Procedure and four labelled Appeal Procedure. The separate ADD roles distinguish the specialized anti-doping forum from Olympic ad hoc review.

Specific contracts or written submissions are distinct from standardized participation and eligibility undertakings. Neither category establishes individual negotiation. Procedural acceptance is separately identified where it supplies the articulated route in the absence of a separately established instrument; routine signing of an Order of Procedure alongside a specific rule is not counted again.

Locations refer to numbered paragraphs where supplied, otherwise the jurisdiction section or specified part of the source. The interpretive notes flag material distinctions; an empty note does not mean that jurisdiction was uncontested or accepted. No missing outcome is inferred from the database result of the appeal.

Category definitions

Code	Category
G	Statutes and constitutions
R	Dispute-resolution and transfer regulations
D	Disciplinary and ethics instruments
E	Competition, selection and admission instruments
A	Anti-doping rules and codes
L	National anti-doping legislation
O	Olympic Charter
C	Specific contractual or written submission instruments
W	Participation and eligibility undertakings
P	Procedural acceptance as articulated route

The P category records conduct as a route, rather than an additional document family. The underlying R55/PILA provisions govern the consequences of acceptance or a late objection; they are not treated as a sports-body authorization.

Within-role counts

Each value counts decisions containing the category within the stated adjudicatory role. Each decision is counted once per category, but may appear in several categories. The column headings give the number of decisions in each role; category totals therefore need not equal those denominators.

Category	Appeal (132)	Direct sole-instance (29)	Olympic ad hoc (7)	ADD (4)
Statutes and constitutions	88	0	0	0
Dispute-resolution and transfer regulations	9	0	0	0
Disciplinary and ethics instruments	13	0	0	0
Competition, selection and admission instruments	9	0	0	0
Anti-doping rules and codes	26	27	0	4
National anti-doping legislation	3	0	0	0
Olympic Charter	6	0	7	3
Specific contractual or written submission instruments	15	2	0	0
Participation and eligibility undertakings	3	0	0	0
Procedural acceptance as articulated route	8	0	0	0

Case-level register

No.	Decision and source	Database label	Adjudicatory role	Code s	Specific instruments	Paragraphs or section	Interpretive note
1	2013/A/3364	Appeal Procedure	Appeal	G	FIFA Statutes: provision for appeal to CAS	Jurisdiction section	—
2	2014/A/3639	Appeal Procedure	Appeal	A, P	NADA Anti-Doping Rules 13.2; R55 and PILA 186(2)	39–52	Regulatory entitlement left undecided; late objection precluded. The NADA provision was not established as a jurisdictional source.
3	2014/A/3695	Appeal Procedure	Appeal	G	FIFA Statutes: provision for appeal to CAS	Jurisdiction section	—

No.	Decision and source	Database label	Adjudicatory role	Codes	Specific instruments	Paragraphs or section	Interpretive note
4	2014/A/3786	Appeal Procedure	Appeal	A	ICF Anti-Doping Rules 13.2 and 13.1.1	Jurisdiction section	WADA appellate entitlement; STJD role distinguished from CAS role.
5	2014/A/3787	Appeal Procedure	Appeal	A	ICF Anti-Doping Rules 13.2 and 13.1.1	Jurisdiction section	WADA appellate entitlement; STJD role distinguished from CAS role.
6	2015/A/3889	Appeal Procedure	Appeal	G, R	RFU Statutes 47(1); Dispute Resolution Regulations 53(2)	5.1–5.8	Agent licence connects appellant to RFU rules; Order of Procedure confirms.
7	2014/A/3565	Appeal Procedure	Appeal	P	Unreserved Order of Procedure; R47	47–49	FIFA jurisdiction is a merits issue. FIFA Statutes 67 appears in admissibility, not as the expressly identified source in this section.
8	2014/A/3759-PA	Appeal Procedure	Appeal	E, G, P	IAAF Hyperandrogenism Regulations 7.2; AFI Rules I and XXXII; express and implicit submission	419–436	Regulatory routes tested; jurisdiction rests on party acceptance; distinguish IAAF and AFI.
9	2015/A/3879	Appeal Procedure	Appeal	G	FIQ Statutes V.5.1 and III.3.2(c)	78–85	Both parties are FIQ members.
10	2015/A/4174	Appeal Procedure	Appeal	G, E, W	ITU Constitution 41; Competition Rules 13.2(c); Athletes Agreement 13	Jurisdiction section	—
11	2014/A/3561 & 3614	Appeal Procedure	Appeal	A, W	IAAF Rule 42; signed doping-control submission forms	Jurisdiction section	Regulatory and written submission grounds require separate treatment.
12	2015/A/3970	Appeal Procedure	Appeal	A	Turkish Anti-Doping Rules 13.2; IAAF Rule 42.4	Jurisdiction section	Direct CAS route rejected for the party/event category; no default appellate jurisdiction.
13	2015/A/3971	Appeal Procedure	Appeal	A	Turkish Anti-Doping Rules 13.2; IAAF Rule 42.4	Jurisdiction section	Direct CAS route rejected for the party/event category; no default appellate jurisdiction.
14	2015/A/4024	Appeal Procedure	Appeal	A	Turkish Anti-Doping Rules 13.2; IAAF Rule 42.4	Jurisdiction section	Direct CAS route rejected for the party/event category; no default appellate jurisdiction.
15	2015/A/3910	Appeal Procedure	Appeal	C	Employment contract arbitration clause	Jurisdiction section	Interpretation of the parties' dispute-resolution clause, including succession; the employment subject matter does not itself identify the jurisdictional source.
16	2014/A/3868	Appeal Procedure	Appeal	A	NADA Anti-Doping Rules 13.2.3	28–36	WADA right to appeal national appellate decision.
17	2014/A/3869	Appeal Procedure	Appeal	A	NADA Anti-Doping Rules 13.2.3	28–36	WADA right to appeal national appellate decision.
18	2015/A/3959	Appeal Procedure	Appeal	C, G	Mandato clause FIFTH; FIFA Statutes 67	Jurisdiction section	Contractual route and statutory route analysed separately for two appellants.

No.	Decision and source	Database label	Adjudicatory role	Codes	Specific instruments	Paragraphs or section	Interpretive note
19	2015/A/3896	Appeal Procedure	Appeal	G	FIFA Statutes: provision for appeal to CAS	Jurisdiction section	—
20	2015/A/4213	Appeal Procedure	Appeal	G	FIFA Statutes: provision for appeal to CAS	Jurisdiction section	—
21	2014/A/3852	Appeal Procedure	Appeal	G, R	FIFA Statutes 66; RSTP 24	63–74	Participation in DRC proceeding supplies appellate connection.
22	2012/A/2877	Appeal Procedure	Appeal	G	FFIRI Statutes 65(1); FIFA Statutes 67(1)	38–50	Neither provision supplies a route for the domestic decision in question.
23	2015/A/3946	Appeal Procedure	Appeal	G, R	FUR Statutes 46–47; Dispute Resolution Regulations 53	52–57	Direct review of DRC decision distinguished from review of PSC decision.
24	2015/A/4254	Appeal Procedure	Appeal	P	Withdrawal of objection; R55 and PILA 186(2)	43–51	Late objection and express withdrawal by different respondents.
25	2015/A/4195	Appeal Procedure	Appeal	G	FIFA Statutes: provision for appeal to CAS	Jurisdiction section	—
26	2015/A/4255	Appeal Procedure	Appeal	G, D	FIG Statutes 21 and 43.1; Code of Discipline 33	60–64	—
27	2015/A/4083	Appeal Procedure	Appeal	G, R	FIFA Statutes 66(1); RSTP 24(2)	81–83	—
28	2014/A/3776	Appeal Procedure	Appeal	G, E	FIFA Statutes 66–67; Admission Regulations 3(1)(s)	Jurisdiction section	Applicant for membership; ordinary membership clause and admission undertaking distinguished.
29	2015/A/4288	Appeal Procedure	Appeal	C	2013 Contract article 14	67–72	Express appeal from BAT award confirmed by both parties.
30	2015/A/4335	Appeal Procedure	Appeal	G	FIFA Statutes: provision for appeal to CAS	Jurisdiction section	—
31	2016/A/4441	Appeal Procedure	Appeal	G	FIFA Statutes: provision for appeal to CAS	Jurisdiction section	—
32	2015/A/4215	Appeal Procedure	Appeal	G, A	FIFA Statutes 67.5; FIFA Anti-Doping Regulations 75 and 81	121–125 and following	FIFA right to appeal; domestic remedies assessed.
33	2016/A/4615	Appeal Procedure	Appeal	C, A	Substantial Assistance Agreement 12; WADC 13.1–13.2	5.3–5.6	—
34	2015/A/4280	Appeal Procedure	Appeal	G	FIFA Statutes: provision for appeal to CAS	Jurisdiction section	—

No.	Decision and source	Database label	Adjudicatory role	Code s	Specific instruments	Paragraphs or section	Interpretive note
35	2015/A/4328	Appeal Procedure	Appeal	G, D	GFA Statutes 68; Disciplinary Code 91	73–76	—
36	2015/A/4351	Appeal Procedure	Appeal	D	LFF Disciplinary Code 92	Jurisdiction section	Appeal Committee decision is the relevant trigger.
37	2016/A/4703	Appeal Procedure	Appeal	C	Arbitration Agreement 12	Jurisdiction section	—
38	2016/A/4657	Appeal Procedure	Appeal	E, C, O	AI selection policy; OCI selection agreement; correspondence; Olympic Charter 61.2	41–49	Claimed routes tested and rejected; no inherent sport-wide jurisdiction.
39	OG AD 16/001	Ad hoc Procedure	Olympic ADD	A, O	CAS Olympic ADD Rules 1; IOC Anti-Doping Rules 8.1; Olympic Charter delegation	Jurisdiction section	Olympic ADD application, not a general ad hoc appeal; no referred ADRV.
40	2015/A/4172	Appeal Procedure	Appeal	G	FUR Statutes 46.1; FIFA Statutes 67.1	40–48 and following	Standing distinguished from competence; affiliation/incorporation analysis.
41	2015/A/4309	Appeal Procedure	Appeal	G	FAI Statutes 2.8.1.1.5	52–58	Statutory appeal from appropriate highest FAI authority; disciplinary subject does not change instrument type.
42	2015/A/4266	Appeal Procedure	Appeal	G	FIFA Statutes: provision for appeal to CAS	Jurisdiction section	—
43	2016/O/4454	Ordinary Procedure	Direct sole-instance	A	IAAF Competition Rules 38.3 and 38.19	Jurisdiction section	Direct sole-instance proceeding; stakeholder consent also considered.
44	2016/O/4455	Ordinary Procedure	Direct sole-instance	A	IAAF Competition Rules 38.3 and 38.19	Jurisdiction section	Direct sole-instance proceeding; stakeholder consent also considered.
45	2016/O/4456	Ordinary Procedure	Direct sole-instance	A	IAAF Competition Rules 38.3 and 38.19	Jurisdiction section	Direct sole-instance proceeding; stakeholder consent also considered.
46	2016/O/4457	Ordinary Procedure	Direct sole-instance	A	IAAF Competition Rules 38.3 and 38.19	Jurisdiction section	Direct sole-instance proceeding; stakeholder consent also considered.
47	2016/O/4465	Ordinary Procedure	Direct sole-instance	A	IAAF Competition Rules 38.3 and 38.19	Jurisdiction section	Direct sole-instance proceeding; stakeholder consent also considered.
48	2016/A/4654	Appeal Procedure	Appeal	G, E	FIFA Statutes 58; World Cup Regulations 14(3)	62–66 and following	Hierarchy between statutory appeal and competition finality provision examined.
49	2016/O/4504	Ordinary Procedure	Direct sole-instance	A	IAAF Competition Rules 38.3	Jurisdiction section	Direct reference following unavailable national hearing; appeal procedural rules do not make this an appeal from a prior award.

No.	Decision and source	Database label	Adjudicatory role	Code s	Specific instruments	Paragraphs or section	Interpretive note
50	2016/O/4702	Ordinary Procedure	Direct sole-instance	A	IAAF Competition Rules 38.3	Jurisdiction section	Direct reference following unavailable national hearing; appeal procedural rules do not make this an appeal from a prior award.
51	2016/A/4450	Appeal Procedure	Appeal	G	FIFA Statutes: provision for appeal to CAS	Jurisdiction section	—
52	2016/A/4697	Appeal Procedure	Appeal	P	Express acceptance in submissions, Order of Procedure and hearing	71–72	—
53	2016/A/4477	Appeal Procedure	Appeal	G	FIFA Statutes: provision for appeal to CAS	Jurisdiction section	—
54	2016/O/4575	Ordinary Procedure	Direct sole-instance	A	IAAF Competition Rules 38.3	Jurisdiction section	Direct reference following unavailable national hearing; appeal procedural rules do not make this an appeal from a prior award.
55	2016/A/4480	Appeal Procedure	Direct sole-instance	A	IAAF Competition Rules 38.19	Jurisdiction section	Database labels Appeal Procedure; decision expressly describes direct sole-instance adjudication with stakeholder consent.
56	2016/A/4486	Appeal Procedure	Direct sole-instance	A	IAAF Competition Rules 38.19	Jurisdiction section	Database labels Appeal Procedure; decision expressly describes direct sole-instance adjudication with stakeholder consent.
57	2016/A/4487	Appeal Procedure	Direct sole-instance	A	IAAF Competition Rules 38.19	Jurisdiction section	Database labels Appeal Procedure; decision expressly describes direct sole-instance adjudication with stakeholder consent.
58	2016/A/4655	Appeal Procedure	Direct sole-instance	A	IAAF Competition Rules 38.19	Jurisdiction section	Database labels Appeal Procedure; decision expressly describes direct sole-instance adjudication with stakeholder consent.
59	2016/A/4777	Appeal Procedure	Appeal	A	IOC Anti-Doping Rules 12.2; Olympic ADD Rules 21	102–103	Present proceeding is an appeal; delegation discussed later concerns prior ADD.
60	2016/O/4853	Ordinary Procedure	Direct sole-instance	A	IAAF Competition Rules 38.3	Jurisdiction section	Direct reference following unavailable national hearing; appeal procedural rules do not make this an appeal from a prior award.
61	2016/O/4854	Ordinary Procedure	Direct sole-instance	A	IAAF Competition Rules 38.3	Jurisdiction section	Direct reference following unavailable national hearing; appeal procedural rules do not make this an appeal from a prior award.
62	2016/A/4473	Appeal Procedure	Appeal	G	FIFA Statutes: provision for appeal to CAS	Jurisdiction section	—

No.	Decision and source	Database label	Adjudicatory role	Codes	Specific instruments	Paragraphs or section	Interpretive note
63	2016/A/4700	Appeal Procedure	Appeal	A	RUSADA Anti-Doping Rules 13.2.3	35 and following	—
64	2016/O/4883	Ordinary Procedure	Direct sole-instance	A	IAAF Competition Rules 38.3	Jurisdiction section	Direct reference following unavailable national hearing; appeal procedural rules do not make this an appeal from a prior award.
65	2016/A/4651	Appeal Procedure	Appeal	G	CONCACAF Statutes 52 and 54	30 and following	—
66	2016/A/4817-IA	Appeal Procedure	Appeal	A	IAAF Anti-Doping Rules 42.2–42.5	71–74	—
67	2016/O/4682	Ordinary Procedure	Direct sole-instance	A	IAAF Competition Rules 38.3	Jurisdiction section	Direct reference following unavailable national hearing; appeal procedural rules do not make this an appeal from a prior award.
68	2016/O/4683	Ordinary Procedure	Direct sole-instance	A	IAAF Competition Rules 38.3	Jurisdiction section	Direct reference following unavailable national hearing; appeal procedural rules do not make this an appeal from a prior award.
69	2014/A/3838	Appeal Procedure	Appeal	G	FIFA Statutes: provision for appeal to CAS	Jurisdiction section	—
70	2017/A/4949	Appeal Procedure	Appeal	A	IAAF Rule 42	61–65	Rule 38.3 at 79–84 concerns the earlier tribunal, not present appellate authority.
71	2017/O/5039	Ordinary Procedure	Direct sole-instance	A	IAAF Competition Rules 38.3	Jurisdiction section	Direct reference following unavailable national hearing; appeal procedural rules do not make this an appeal from a prior award.
72	2017/O/4978	Ordinary Procedure	Direct sole-instance	A	IAAF Competition Rules 38.3	Jurisdiction section	Direct reference following unavailable national hearing; appeal procedural rules do not make this an appeal from a prior award.
73	2016/A/4787	Appeal Procedure	Appeal	G	UEFA Statutes 62	92–99	Panel accepts jurisdiction over non-member JFA as directly affected party; earlier text denying this is UEFA's submission.
74	2016/A/4839	Appeal Procedure	Appeal	A, O, P	Beijing ADR 12.2; Olympic Charter; explicit acceptance of CAS jurisdiction	292–296	Beijing ADR disputed; panel records express acceptance and Olympic Charter route.
75	2017/A/4947	Appeal Procedure	Appeal	G	RFF Statutes 57.4 and 57.6	Jurisdiction section	—
76	2017/A/5057	Appeal Procedure	Appeal	G	NOSC Statute 23.2	167–169	Respondent abandons objection; suspension of federation covered.

No.	Decision and source	Database label	Adjudicatory role	Codes	Specific instruments	Paragraphs or section	Interpretive note
77	2016/A/4770	Appeal Procedure	Appeal	E, W	IPC Handbook 2.8; Paralympic Eligibility Code Form	Jurisdiction section	Participation/accreditation conditions tested; do not label this Olympic Charter.
78	2017/A/5065	Appeal Procedure	Appeal	C	Employment Contract 6.3 and 10.2	Jurisdiction section	Clause naming a domestic arbitration council tested; not automatically a CAS agreement.
79	2017/A/5133	Appeal Procedure	Appeal	R	FFU DRC Regulations 60	39 and following	—
80	2017/A/5072	Appeal Procedure	Appeal	C	Contract 11 and incorporated BAT Rules	43–55	No agreement for CAS appeal found; prior cases' agreements are not this case's source.
81	2017/A/5128	Appeal Procedure	Appeal	C, G	Contract 4.6; BFF Statutes; FIFA Statutes 59.3	50–64	Domestic Belarus dispute; claimed CAS routes rejected.
82	2017/A/5333	Appeal Procedure	Appeal	G	MFA Statutes 3(ii)	44–46	—
83	OG 18/004	Ad hoc Procedure	Olympic ad hoc	O	Olympic Charter 61.2; CAS Ad Hoc Rules 1	Jurisdiction section	General Olympic ad hoc forum; temporal and dispute-scope requirements recorded separately.
84	OG 18/005	Ad hoc Procedure	Olympic ad hoc	O	Olympic Charter 61.2; CAS Ad Hoc Rules 1	Jurisdiction section	General Olympic ad hoc forum; temporal and dispute-scope requirements recorded separately.
85	OG AD 18/003-PA	Ad hoc Procedure	Olympic ADD	O, A	Olympic Charter 59.2.4; IOC Anti-Doping Rules 8.1.1; CAS Olympic ADD Rules 1	Jurisdiction section	Olympic Anti-Doping Division partial decision; database ad hoc label retained separately.
86	OG AD 18/005-PA	Ad hoc Procedure	Olympic ADD	O, A	Olympic Charter 59.2.4; IOC Anti-Doping Rules 8.1.1; CAS Olympic ADD Rules 1	Jurisdiction section	Olympic Anti-Doping Division partial decision; database ad hoc label retained separately.
87	2017/A/5187	Appeal Procedure	Appeal	G	FIFA Statutes: provision for appeal to CAS	Jurisdiction section	—
88	2017/O/5330	Ordinary Procedure	Direct sole-instance	A	IAAF Competition Rules 38.3	Jurisdiction section	Direct reference following unavailable national hearing; appeal procedural rules do not make this an appeal from a prior award.
89	2017/A/5054	Appeal Procedure	Appeal	G	FIFA Statutes: provision for appeal to CAS	Jurisdiction section	—
90	2017/O/5376	Ordinary Procedure	Direct sole-instance	A	IAAF Competition Rules 38.3	Jurisdiction section	Direct reference following unavailable national hearing; appeal procedural rules do not make this an appeal from a prior award.

No.	Decision and source	Database label	Adjudicatory role	Code s	Specific instruments	Paragraphs or section	Interpretive note
91	2017/O/5389	Ordinary Procedure	Direct sole-instance	A	IAAF Competition Rules 38.3	Jurisdiction section	Direct reference following unavailable national hearing; appeal procedural rules do not make this an appeal from a prior award.
92	2017/O/5331	Ordinary Procedure	Direct sole-instance	A	IAAF Competition Rules 38.3	Jurisdiction section	Direct reference following unavailable national hearing; appeal procedural rules do not make this an appeal from a prior award.
93	2017/O/5332	Ordinary Procedure	Direct sole-instance	A	IAAF Competition Rules 38.3	Jurisdiction section	Direct reference following unavailable national hearing; appeal procedural rules do not make this an appeal from a prior award.
94	2017/A/5459	Appeal Procedure	Appeal	G, C	ICMG Charter VII.4; President's letter of 5 December 2017	77–91	Letter operates as accepted offer to arbitrate, but is not itself appealable decision.
95	2017/A/5421	Appeal Procedure	Appeal	D	NBB Regulation on Disciplinary and Dispute Proceedings A.66	32–35	—
96	2017/A/5131	Appeal Procedure	Appeal	G, D	FIDE Statutes 13.1; Code of Ethics 4.5	Jurisdiction section	—
97	2017/A/5209	Appeal Procedure	Appeal	L, A, G	Romanian Law 227/2006, 76; Appeal Commission Regulations 33; RKCF Statutes 84	71–76	Legislation read with appeal regulations and federation statute.
98	2017/A/5200	Appeal Procedure	Appeal	G	IPF Bylaws 31.1	89–92 and following	Expressly includes other individuals or organizations; non-member status not dispositive.
99	2018/A/5658	Appeal Procedure	Appeal	G	LFA Statutes 87	40–43	—
100	2017/A/5324	Appeal Procedure	Appeal	G, E	FIFA Statutes 58; World Cup competition regulations	Jurisdiction section	Scope and hierarchy of finality/appeal provisions examined.
101	2017/A/5356	Appeal Procedure	Appeal	G, E	FIFA Statutes 58; World Cup competition regulations	Jurisdiction section	Scope and hierarchy of finality/appeal provisions examined.
102	2018/A/5586	Appeal Procedure	Appeal	G	FIFA Statutes: provision for appeal to CAS	Jurisdiction section	—
103	2018/A/5664	Appeal Procedure	Appeal	G	FIFA Statutes: provision for appeal to CAS	Jurisdiction section	—
104	2018/A/5624	Appeal Procedure	Appeal	G	FIFA Statutes: provision for appeal to CAS	Jurisdiction section	—
105	2018/A/5628	Appeal Procedure	Appeal	G	FIFA Statutes: provision for appeal to CAS	Jurisdiction section	—

No.	Decision and source	Database label	Adjudicatory role	Codes	Specific instruments	Paragraphs or section	Interpretive note
106	2018/A/5661	Appeal Procedure	Appeal	G	FIFA Statutes: provision for appeal to CAS	Jurisdiction section	—
107	2018/A/5647	Appeal Procedure	Appeal	G	FIFA Statutes: provision for appeal to CAS	Jurisdiction section	—
108	2017/A/5475	Appeal Procedure	Appeal	G	EOC Statutes 51.4	106–108	Actual panel analysis appears after party positions.
109	2018/A/5520	Appeal Procedure	Appeal	A	IAAF Competition Rules 42	87–90	Current appeal from first-instance arbitrator; Rule 38.3 concerns earlier proceeding.
110	2018/A/5854	Appeal Procedure	Appeal	G, D	FIFA Statutes 58; FIFA Disciplinary Code 64	Jurisdiction section	—
111	2017/O/5398	Ordinary Procedure	Direct sole-instance	A	IAAF Competition Rules 38.3	Jurisdiction section	Direct reference following unavailable national hearing; appeal procedural rules do not make this an appeal from a prior award.
112	2018/A/5782	Appeal Procedure	Appeal	G, R	UAE FA statutes; AFC and FIFA statutes; UAE FA Arbitration Tribunal Regulations 7(3) and 50	67–73	Domestic appeal route must be expressly provided; source not inferred from transfer subject.
113	2018/A/5659	Appeal Procedure	Appeal	G	FIFA Statutes: provision for appeal to CAS	Jurisdiction section	—
114	2018/O/5822	Ordinary Procedure	Direct sole-instance	A	IAAF ADR 2018, 21.3; IAAF Competition Rules 2016, 38.3	57–63 and following	Transitional provision determines applicable direct-reference rule.
115	2018/O/5754	Ordinary Procedure	Direct sole-instance	C	Specific Arbitration Agreement	27, 99–100	Direct sole-instance case assigned to Ordinary Division with appeal procedural rules.
116	2018/A/5945	Appeal Procedure	Appeal	G, R	FUR Statutes 47; Dispute Resolution Regulations 53	Jurisdiction section	—
117	2018/A/5853	Appeal Procedure	Appeal	G, A	FIFA Statutes 57–58.5; FIFA ADR 80–81	82–86 and following	National anti-doping tribunal within delegated disciplinary structure.
118	2017/A/5498	Appeal Procedure	Appeal	O, P	Olympic Charter 61.2; respondent's express acceptance	38 and following	Respondent questions Charter coverage but accepts this panel's jurisdiction.
119	2019/A/6330	Appeal Procedure	Appeal	G, C	World Skate Statutes 2(4) and 3; written submission agreement	47–53	Statutory personal scope doubtful; express agreement supplies the connection.
120	2018/A/5746	Appeal Procedure	Appeal	G, D	FIFA Statutes 58; Disciplinary Code 128	123 and following	—

No.	Decision and source	Database label	Adjudicatory role	Code s	Specific instruments	Paragraphs or section	Interpretive note
121	2018/A/5595	Appeal Procedure	Appeal	G, D	GFA Statutes 43.1; Disciplinary Code 91	49–50 and following	—
122	2018/A/6064	Appeal Procedure	Appeal	G, C	FIFA Statutes 58(1); contract article XV	53–54 and following	—
123	2019/A/6131	Appeal Procedure	Appeal	G	FIFA Statutes: provision for appeal to CAS	Jurisdiction section	—
124	2018/A/6052	Appeal Procedure	Appeal	G, R	FKF Constitution; FIFA Statutes 58; SDT Draft Rules 28(b)	46–65	Draft rules not shown to be in force; no CAS route established.
125	2018/A/5545	Appeal Procedure	Appeal	G	EOC Decree 242/2017, 51(4)	82–85	—
126	2018/A/5868	Appeal Procedure	Appeal	G	IHF Statutes 23	74 and following	—
127	2018/A/6025	Appeal Procedure	Appeal	A	Czech anti-doping Directive 13; WADC 13.1–13.2.2	53–69	Panel's specific denial-of-justice reasoning; distinguish ordinary national appeal rule.
128	2019/A/6112	Appeal Procedure	Appeal	L	Romanian Anti-Doping Law 76(1)	43–45	—
129	2019/A/6225	Appeal Procedure	Appeal	O, G	Olympic Charter 61.2; IAAF Constitution 84.3	Jurisdiction section	Different respondents and different proposed clauses must be kept separate.
130	2019/A/6274	Appeal Procedure	Appeal	O	Olympic Charter 61.2	51 and following	Offer-to-world interpretation disputed; no automatic agreement for every claimant.
131	2018/A/5982	Appeal Procedure	Appeal	G	SFA Statutes 66.4	51–54 and following	—
132	2019/A/6636	Appeal Procedure	Appeal	E	RBF Championship Regulations 94.7	62–72	Alternative choice of NCSA or CAS; licensing subject is not a distinct licensing arbitration provision.
133	2020/A/6781	Appeal Procedure	Appeal	A, G	FIS Anti-Doping Rules 13.2.3; FIS Statutes 57.4	Jurisdiction discussion in termination order	Provisional suspension appeal; regulations can contain arbitration clause.
134	2019/A/6226	Appeal Procedure	Appeal	L, A	Spanish Anti-Doping Act 40.6; WADC 13.2.3	83–95	Licence and statutory incorporation; agency's WADC commitment assessed separately.
135	2019/A/6312	Appeal Procedure	Appeal	G	FIFA Statutes: provision for appeal to CAS	Jurisdiction section	—

No.	Decision and source	Database label	Adjudicatory role	Code s	Specific instruments	Paragraphs or section	Interpretive note
136	2018/O/5830	Ordinary Procedure	Direct sole-instance	C	MoU and exchanged written communications	160–161 and following	Unsigned MoU supported by correspondence; signatures not the only evidence of agreement.
137	2020/A/6820	Appeal Procedure	Appeal	G	FIFA Statutes: provision for appeal to CAS	Jurisdiction section	—
138	2019/A/6569	Appeal Procedure	Appeal	G	FIFA Statutes 58(1)	65–68	Employment contract jurisdiction is analysed as FIFA competence on merits, not counted as CAS authorization.
139	2020/A/6933	Appeal Procedure	Appeal	G	FIFA Statutes: provision for appeal to CAS	Jurisdiction section	—
140	2020/A/6986	Appeal Procedure	Appeal	A	RUSADA Anti-Doping Rules 13.2.2.1	61–66	National-level athlete category expressly routed to CAS by applicable rules.
141	2020/A/6987	Appeal Procedure	Appeal	A	RUSADA Anti-Doping Rules 13.2.2.1	61–66	National-level athlete category expressly routed to CAS by applicable rules.
142	2020/A/6988	Appeal Procedure	Appeal	A	RUSADA Anti-Doping Rules 13.2.2.1	61–66	National-level athlete category expressly routed to CAS by applicable rules.
143	2021/A/8031	Appeal Procedure	Appeal	G	FINA Constitution C12.13	75–79	Executive decision must first go to Bureau; no direct route established.
144	OG 20/002	Ad hoc Procedure	Olympic ad hoc	O	Olympic Charter 61.2; CAS Ad Hoc Rules 1	Jurisdiction section	General Olympic ad hoc forum; temporal and dispute-scope requirements recorded separately.
145	OG 20/006 & 008	Ad hoc Procedure	Olympic ad hoc	O	Olympic Charter 61.2; CAS Ad Hoc Rules 1	Jurisdiction section	General Olympic ad hoc forum; temporal and dispute-scope requirements recorded separately.
146	OG 20/010 & 011	Ad hoc Procedure	Olympic ad hoc	O	Olympic Charter 61.2; CAS Ad Hoc Rules 1	Jurisdiction section	General Olympic ad hoc forum; temporal and dispute-scope requirements recorded separately.
147	OG 22/002	Ad hoc Procedure	Olympic ad hoc	O	Olympic Charter 61.2; CAS Ad Hoc Rules 1	Jurisdiction section	General Olympic ad hoc forum; temporal and dispute-scope requirements recorded separately.
148	OG 22/008 & 009 & 010	Ad hoc Procedure	Olympic ad hoc	O	Olympic Charter 61.2; CAS Ad Hoc Rules 1	Jurisdiction section	General Olympic ad hoc forum; temporal and dispute-scope requirements recorded separately.
149	2021/A/8077	Appeal Procedure	Appeal	D, G	WKF Disciplinary and Ethics Code 26; Statutes 21.9	37–39	—
150	2021/ADD/14	Anti-doping Procedure (first instance)	CAS ADD	A	IBU Anti-Doping Rules 8.1.2.1; CAS ADD Rules A2	188–195	Registered athlete; first-instance delegation and incorporation.

No.	Decision and source	Database label	Adjudicatory role	Code s	Specific instruments	Paragraphs or section	Interpretive note
151	2021/A/8403	Appeal Procedure	Appeal	G, D	SAFA Statutes 11.2.4 and 73; Disciplinary Code 74, 127, 129; FIFA Statutes	83–93	No route for decision refusing review; recognition of awards differs from authorization of appeals.
152	2021/A/8413	Appeal Procedure	Appeal	G	SFA Statutes 66 and 68	62–66	—
153	2021/A/7636	Appeal Procedure	Appeal	G	FIFA Statutes: provision for appeal to CAS	Jurisdiction section	—
154	2019/A/6594	Appeal Procedure	Appeal	G	FIFA Statutes: provision for appeal to CAS	Jurisdiction section	—
155	2021/A/8417	Appeal Procedure	Appeal	G, D	IWUF Constitution 36.8–36.10 / 38.9; Code of Ethics J.3	55–62 and following	Version and final-decision conditions analysed.
156	2021/A/7915	Appeal Procedure	Appeal	G	FIFA Statutes: provision for appeal to CAS	Jurisdiction section	—
157	2021/A/8008	Appeal Procedure	Appeal	G	FIFA Statutes: provision for appeal to CAS	Jurisdiction section	—
158	2021/A/7775	Appeal Procedure	Appeal	G, C	FIFA Statutes 58(1); Employment Contract XI.4	82–85 and following	—
159	2022/A/8621	Appeal Procedure	Appeal	G, C	FIFA Statutes 57(1); employment Contract 8(2); image-rights agreement 12	85–92	Employment claims have CAS route; separate image-rights jurisdiction tested independently.
160	2021/A/7996	Appeal Procedure	Appeal	G	EFA Statutes 45 and 46	62–77	Direct route absent; prior domestic appeal required.
161	2022/A/8700	Appeal Procedure	Appeal	A	POLADA Anti-Doping Rules 13.2	39–48 and following	Athlete category determines forum; entitled persons and appellate body distinguished.
162	2022/A/9351	Appeal Procedure	Appeal	G	FINA Constitution C12.13.3 (2021)	59 and following	—
163	2023/A/9518	Appeal Procedure	Appeal	C	Arbitration Agreement of 13 March 2023	60 and following	—
164	2023/O/9401	Ordinary Procedure	Direct sole-instance	A	WA ADR 1.7.2(b); IAAF Competition Rules 38.3	93–97	Transitional rule supports direct sole-instance referral.
165	2022/A/8914	Appeal Procedure	Appeal	G	ITF Constitution 33(c)	98–100	Jurisdiction over ITF; no jurisdiction over absent separate entity Tennis Europe.

No.	Decision and source	Database label	Adjudicatory role	Code s	Specific instruments	Paragraphs or section	Interpretive note
166	2023/A/9477	Appeal Procedure	Appeal	G	FIFA Statutes: provision for appeal to CAS	Jurisdiction section	—
167	2023/A/9611	Appeal Procedure	Appeal	G	FIFA Statutes: provision for appeal to CAS	Jurisdiction section	—
168	2023/A/10093	Appeal Procedure	Appeal	P	IOC acceptance in present arbitration	38–39	No specific sports-body arbitration provision identified in jurisdiction section; the respondent's identity alone does not establish an Olympic Charter basis.
169	2023/A/10168	Appeal Procedure	Appeal	R, D	HFF Rules Governing Procedure before Judiciary Bodies; Disciplinary Code 25(5)	66–67	—
170	2022/A/8664	Appeal Procedure	Appeal	G	CAF Statutes 48(1) and 48(3)	50–57	Internal exhaustion assessed before CAS review.
171	2022/A/9053	Appeal Procedure	Appeal	D	Tennis Anti-Corruption Program I.1 (2021)	63–65	—
172	2023/A/9757	Appeal Procedure	Appeal	O	Olympic Charter 61	296–298	—
